# The Hygienic Criterion of Air; Its Capacity for Drying the Skin

**Published:** 1919-01-18

**Authors:** 


### HUMIDITY AND HEALTH.

### The Hygienic Criterion of Air; Its Capacity for Drying the Skin.

By most people humid air is considered unhealthy air. But there seems to be little ground for holding that damp is bad for the respiratory organs.

After all, moisture, in some measure, is essential to life. The cells of our body have had a very aquatic past and have still very aquatic predilections, so much so, that the abstraction of even ten per cent. of body water means death, and, as is well known, thirst kills faster than starvation. Indeed, in spite of popular opinion, it is not very obvious why damp air should be unhealthy at all.

Humidity is usually supposed to be specially bad for the lungs, and to damp air are usually ascribed all lung diseases, such as tuberculosis and bronchitis. Nevertheless, as a matter of scientific fact, damp is probably not bad for the lungs, for nature has deliberately moistened the nose and air passages, so that all air, even dry air, is laden with moisture before it reaches the lungs. Moreover, it is well known that bronchitis can be relieved by the steam of a bronchitis kettle, and most sanatorium doctors have noticed that damp weather often soothes the cough of consumptive patients. Nor can even cold damp air hurt the lungs, for nature has also arranged for the heating of the air before it reaches the pulmonary air-cells. Air 14° F. below freezing point if breathed through the nose (as it should be) is warmed to 77° F. by the time it arrives at the throat. The most we can admit is that the cold air may, by abstraction of heat from the nose, reduce the resistance of the nasal mucous membrane to germs; but that is a matter of coldness not of dampness, and against such a possibility we may set the facts that dry air contains more dust and germs than moist air, and that in the United States, where the air is particularly dry, nasal catarrh is especially prevalent.

Damp is pernicious mainly in its physiological relationship to the skin. Active trans-sudation (if we may coin the word) of the water of the tissues through the skin is indubitably essential to health, and health is quickly impaired by any interference with the skin's sudorific function. Now, damp air by its complete or comparative incapacity to evaporate the sensible and insensible moisture of the skin limits trans-sudation. Less water is drunk, and less is evaporated, and it is probable that the subcutaneous tissues, despite some renal compensation, become, so to speak, waterlogged, as is shown by the swelling of a corn on a damp day—a fact that has always seemed to us of striking physiological significance. The result is that the bathing and cleansing of the cells of the body and the excretion of poisons are less efficient, that the circulation of lymph and blood is retarded, and that the great vital functions of assimilation, oxidation, excretion, and secretion are all impaired. The feeling of languor and slackness so often experienced in damp weather is mainly due to less copious trans-sudation and less potent evaporation; and the feeling of vigour and exhilaration produced by dry air is mainly due to the facilitation and acceleration of the passage of water from the tissues of the body to the air.

We reach the conclusion, therefore—a conclusion contrary to popular opinion—that damp has no more bad effects on the lungs than may follow some excess of fluid in the body, and that it is detrimental to health only in so far as it is an impediment to active trans-sudation and evaporation. "In so far as it is an impediment," we say, and the question of how far it is an impediment must be understood to depend not only on the humidity but also on the temperature of the air.

Meteorologists have supplied health resorts with statistics showing the "absolute humidity" of the air, *i.e.* how many grammes of water vapour are contained in a cubic foot of air, or showing the "relative humidity," *i.e.* how much water vapour the air contains compared with the amount it is capable of containing; and hygienists, with an imperfect conception of the physiological effects of damp, have given these statistics great hygienic significance. But from what we have already pointed out it is evident that the hygienic criterion of air is neither its *absolute* humidity, nor its *relative* humidity, but its capacity for drying the skin, and this depends not only on the humidity of the air but also on its temperature. Thus, at 0° C. a cubic metre of air, with a relative humidity of sixty per cent., is able to soak up about 2,000 grammes of water vapour, while at 30° C. the same volume of air, with the same relative humidity, is able to soak up about 12,000 grammes of water vapour, *i.e.* the hotter air has six times the drying capacity of the cooler air. Again, air at 30° C. with a relative humidity of eighty per cent. (very damp, that is to say), will be as drying as perfectly dry air at 3° C.

In any estimate of the hygienic character of damp air, then, we have to consider not only the humidity but the temperature of the air. But there are still other factors that must be taken into consideration. The movement of the air, as is well known, greatly affects its drying capacity. *Ceteris paribus*, air in rapid movement, is much more drying than still air and it is that chiefly which gives sea and mountain breezes their therapeutic value. Again, *ceteris paribus*, the rarer air of high altitudes, is more drying than the denser air at sea level, and this partly explains the benefit derived by residence in high health resorts. The relation of the movement of the air to its drying capacity is of special importance in the matter of indoor ventilation. A movement of indoor air is desirable not only to keep the air pure but to increase its drying capacity.

When we warm indoor air we increase its drying capacity, and unless we render it *too* drying, as sometimes happens, that is so far good. But under ordinary domestic conditions of inadequate ventila-

**Humidity and Health—(*continued*).**

tion indoor air quickly becomes laden with moisture from the skins and lungs of living creatures and movement is required to augment its drying capacity quite as much as to restore its purity. It must be particularly noted in this connection that the air whose dryingness is most important, *i.e.* the air under the garments, next to the skin, is just the air most prone to be saturated with moisture, and the air most apt to be stagnant. So long as the intra-garmental air is humid and stagnant, evaporation from the skin will be retarded, no matter how drying the extra-garmental air may be. When one wears an air-tight garment such as a waterproof, one realises how both heat and moisture are retained within it; but in an ill-ventilated room, without perceptible currents of air, even ordinary garments may retain a layer of warm and wet air round the body, and thus impede the outward passage both of moisture and of heat—a double impediment deleterious to all the vital functions. Ventilation, from the point of view of damp, must consider not only house but habiliments, for the extra garments some people wear in wet weather to keep the damp out really keep the damp in.

The general relationships between humidity and the cooling and heating effects of cold and hot air on the body cannot be considered here, nor the effect of moisture in the atmosphere on the penetration and radiation of light and heat; the purpose of this article is simply to emphasise the advantages of air with good drying capacity, and to point out that *dryingness* and not absolute or relative humidity is the best criterion of air regarded from the standpoint of health.

What we need at present, it seems to the writer, are systematic records not of the humidity of the air but of the dryingness of the air in various places and under various conditions. Such records could be easily made by an apparatus designed simply to measure evaporation, and they would be of much greater value for hygienic purposes than the records of relative and absolute humidity at present published by the meteorologists.

